# Development of a Novel Molecular Sensor for Imaging Estrogen Receptor-Coactivator Protein-Protein Interactions

**DOI:** 10.1371/journal.pone.0044160

**Published:** 2012-08-28

**Authors:** Madryn C. Lake, Quang-Dé Nguyen, Simak Ali, Eric O. Aboagye

**Affiliations:** 1 Comprehensive Cancer Imaging Centre, Imperial College London, London, United Kingdom; 2 Department of Surgery and Cancer, Imperial College London, London, United Kingdom; Roswell Park Cancer Institute, United States of America

## Abstract

Anti-estrogens, in particular tissue selective anti-estrogens, have been the bedrock of adjuvant therapy for patients with estrogen receptor alpha (ERα) positive breast cancer. Though current therapies have greatly enhanced patient prognosis, there continues to be an impetus for the development of improved anti-estrogens. ERα is a nuclear receptor transcription factor which activates gene expression through the recruitment of transcriptional coactivator proteins. The SRC family of coactivators, which includes AIB1, has been shown to be of particular importance for ERα mediated transcription. ERα-AIB1 interactions are indicative of gene expression and are inhibited by anti-estrogen treatment. We have exploited the interaction between ERα and AIB1 as a novel method for imaging ERα activity using a split luciferase molecular sensor. By producing a range of ERα ligand binding domain (ER-LBD) and AIB1 nuclear receptor interacting domain (AIB-RID) N- and C-terminal firefly luciferase fragment fusion proteins, constructs which exhibited more than a 10-fold increase in luciferase activity with E2 stimulation were identified. The specificity of the E2-stimulated luciferase activity to ERα-AIB1 interaction was validated through Y537S and L539/540A ER-LBD fusion protein mutants. The primed nature of the split luciferase assay allowed changes in ERα activity, with respect to the protein-protein interactions preceding transcription, to be assessed soon after drug treatment. The novel assay split luciferase detailed in this report enabled modulation of ERα activity to be sensitively imaged *in vitro* and in living subjects and potentially holds much promise for imaging the efficacy of novel ERα specific therapies.

## Introduction

Breast cancer is the most common cancer in women in the Western world and the most common cause of female cancer death worldwide. It is estimated that more than 1 in 9 women in the West will be affected by the disease during their lifetime [1].

Estrogen is a steroid hormone that has been linked to the initiation and progression of breast cancer. As the female sex hormone, estrogen is critical for regulation of the menstrual cycle and the development of female secondary sexual characteristics. However, in addition to these functions, estrogen also exerts a range of more homeostatic effects. Most notably, it protects against cardiovascular disease and helps to maintain bone density [2]. Estrogen exerts its effects through the action of the estrogen receptors α and β (ERα and ERβ), which are members of the large nuclear receptor family of transcription factors that are typically activated upon binding to small lipophilic molecules [3]. Although evidence for the role of ERß in breast cancer remains unclear, the importance of ERα in breast cancer is well-established [4], [5]. ERα is able to regulate gene expression through association with coactivator and corepressor proteins. These proteins act as scaffolds for further protein recruitment to promote transcription complex assembly or the formation of a transcription repressing complex [6]. The canonical mechanism through which ERα regulates gene transcription is by binding to a 13 base pair palindromic estrogen response element (ERE) in the promoters of estrogen responsive genes [7]. However, ERα is also able to regulate the expression of genes which do not contain an ERE through interaction with other transcription factors, in particular AP1 and Sp1 [8], [9]. Indeed, ChIP experiments have suggested that as few as 11% of ERα regulated genes contain an ERE [10].

Approximately two thirds of breast tumors express ERα and therapies which interrupt the estrogen signaling pathway have proven effective for the treatment of this breast cancer subtype. This has been achieved through a number of different methods, including ERα antagonists and down regulators, as well as aromatase inhibitors, which inhibit estrogen biosynthesis. However, because of the range of effects which estrogen exerts in tissues other than the breast and uterus, a complete withdrawal of estrogen can be associated with a range of negative side effects, most notably menopausal symptoms, joint disorders and a loss of bone density [2]. Selective estrogen receptor modulators (SERMs) are a class of compounds, which can overcome this problem; their mixed agonist and antagonist actions in different target tissues offers the potential to block estrogen action in the breast and uterus while maintaining the positive influence of estrogen in bone and the cardiovascular system. Tamoxifen, the prototype SERM, has continued to be one of the most popular treatments for breast cancer for over 30 years because its anti-estrogen action in the breast is balanced by pro-estrogen actions in other tissues: tamoxifen increases bone mineral density and reduces cholesterol, and its use is associated with a reduction in osteoporotic fractures and cardiovascular deaths [11]–[15]. However, although tamoxifen has undoubtedly greatly improved the prognosis of thousands of breast cancer patients, because it acts as a mild estrogen agonist in the uterus, its use is associated with an increased incidence of endometrial cancer, in addition to other negative side effects, such as hot flushes and increased thromboembolism [2], [16]. Because of these limitations, there continues to be much interest in the development of novel SERMs and anti-estrogens.

The mechanisms through which SERMs are able to exert ERα agonist and antagonist properties in different tissues are not fully understood, but their mixed actions are known to require different ERα-coregulator interactions. Tamoxifen bound ERα predominantly recruits coactivator proteins in endometrial cells, but recruits corepressor proteins in breast cancer cells; these different coregulator recruitment profiles enable tamoxifen to stimulate or inhibit transcription in the two cell types [17].

**Figure 1:**
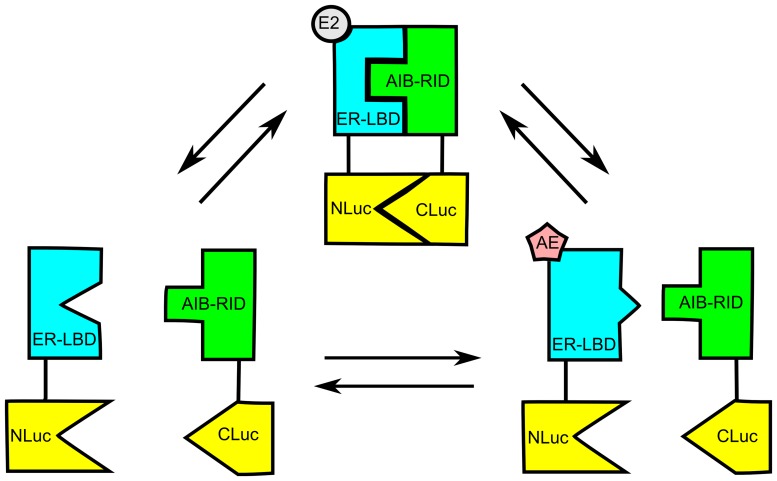
Experimental design of the ERα-AIB1 split luciferase assay. Estrogen (E2) binding promotes ERα-AIB1 interaction and consequent reconstitution of the N- (NLuc) and C-terminal (CLuc) portions of the split firefly luciferase. In the case of anti-estrogens (AE), the interaction may be dependent on the tissue and AE context.

In the development of novel anti-estrogens, the pro- or anti-estrogen action of novel ERα ligands is generally assessed by measuring ERα transcriptional output from either endogenous genes using RT-PCR or through quantification of estrogen regulated reporter genes in genetically modified cell lines [18]. Both of these methods, though undoubtedly powerful and valuable techniques, focus the agonist/antagonist read-out on a select number of ERα promoters when it is commonly recognized that ERα is able to exert genomic effects through a range of different mechanisms [10].

Here we describe a novel method for visualizing ERα activity using a split luciferase assay to report on the ligand induced association between ERα and its coactivator amplified in breast cancer-1 (AIB1). AIB1 (SRC-3) is a member of the steroid receptor coactivator (SRC) family of nuclear receptor coactivators that also include SRC-1 and SRC-2, all of which are capable of interacting with ERα to potentiate its activity [19]. AIB1 is believed to be fundamental to ERα signaling in the development and progression of breast cancer; it is amplified or overexpressed in approximately two thirds of human breast cancers and overexpression of AIB1 in the mammary epithelium of mice leads to the formation of mammary adenocarcinomas, 85% of which express ERα [20], [21]. Laboratory studies have indicated that AIB1 acts as a rate-limiting factor for ERα signaling and knock down of the protein attenuates the E2 stimulated proliferation of breast cancer cells [22], [23]. By imaging the events which are key to gene regulation by ERα, namely the recruitment of transcriptional co-activators, we hope to develop an effective method for measuring the cellular response to ERα modulators *in vitro* and *in vivo*.

**Figure 2:**
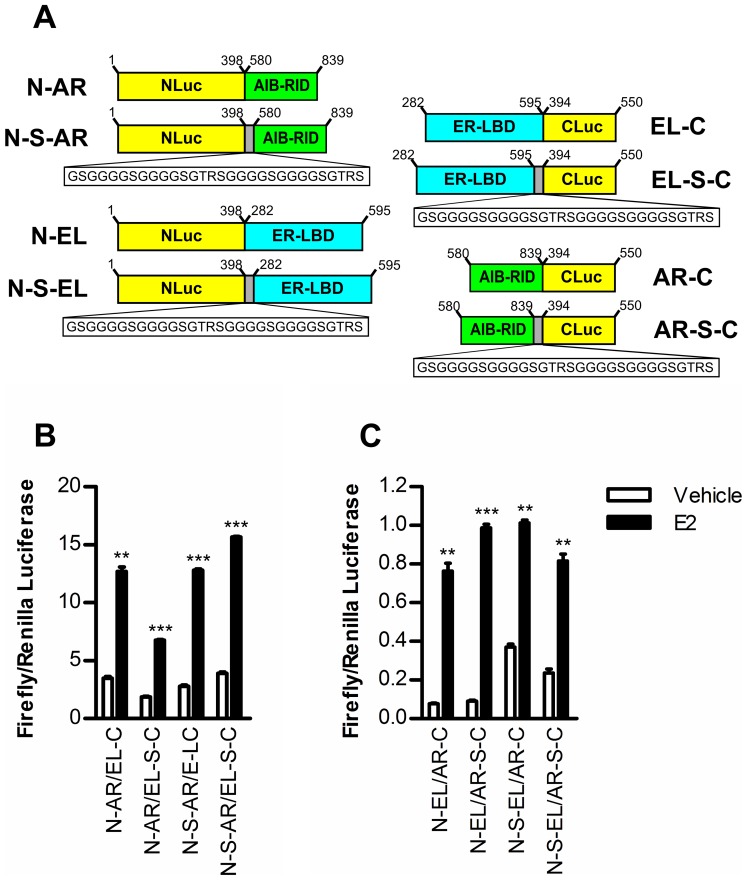
ERα-AIB1 split luciferase construct optimization. (A) Schematic representation of the ERα ligand binding domain (ER-LBD) and AIB1 nuclear receptor interacting domain (AIB-RID) fusion constructs used in this study. The grey box represents a flexible spacer (-S-) with the sequence GSGGGGSGGGGSGTRSGGGGSGGGGSGTRS, where G is glycine, S is serine T is threonine and R is arginine. (B) 293 cells were transiently transfected with the indicated split firefly luciferase constructs and *Renilla* luciferase to control for transfection efficiency. Luciferase activity was determined 48 hours following the addition of 1 µM E2 or vehicle. The bar charts indicate the ratio of firefly luciferase activity relative to *Renilla* luciferase activity (± standard error of the mean (SEM) of triplicates). A t-test was used to determine statistical significance relative to vehicle treatment (*** p≤0.001, **p≤0.01).

Towards dynamic imaging of ERα-AIB1 protein-protein interactions, we have employed a split luciferase assay in which the ERα ligand binding domain (ER-LBD) and AIB1 nuclear receptor interaction domain (AIB-RID) are expressed as fusion proteins with the N-terminal or C-terminal portions of firefly luciferase (NLuc and CLuc, respectively; Figure 1). The split luciferase fragments are enzymatically inactive in isolation, but functional luciferase activity is restored when the N- and C-terminal fragments are brought into close proximity by interaction of the two luciferase fusion partners, thereby enabling the visualization of protein-protein interactions by an increase in luciferase activity [24], [25]. Using this method, we show that ERα interaction with the transcriptional co-regulator AIB1 can be imaged in living subjects as well as *in vitro*. Since differential ERα-AIB1 interactions have been shown to correspond with the tissue specific actions of SERMs [17] it is anticipated that this new method could provide the basis for investigating the tissue-selective action of SERMs *in vivo*.

**Figure 3:**
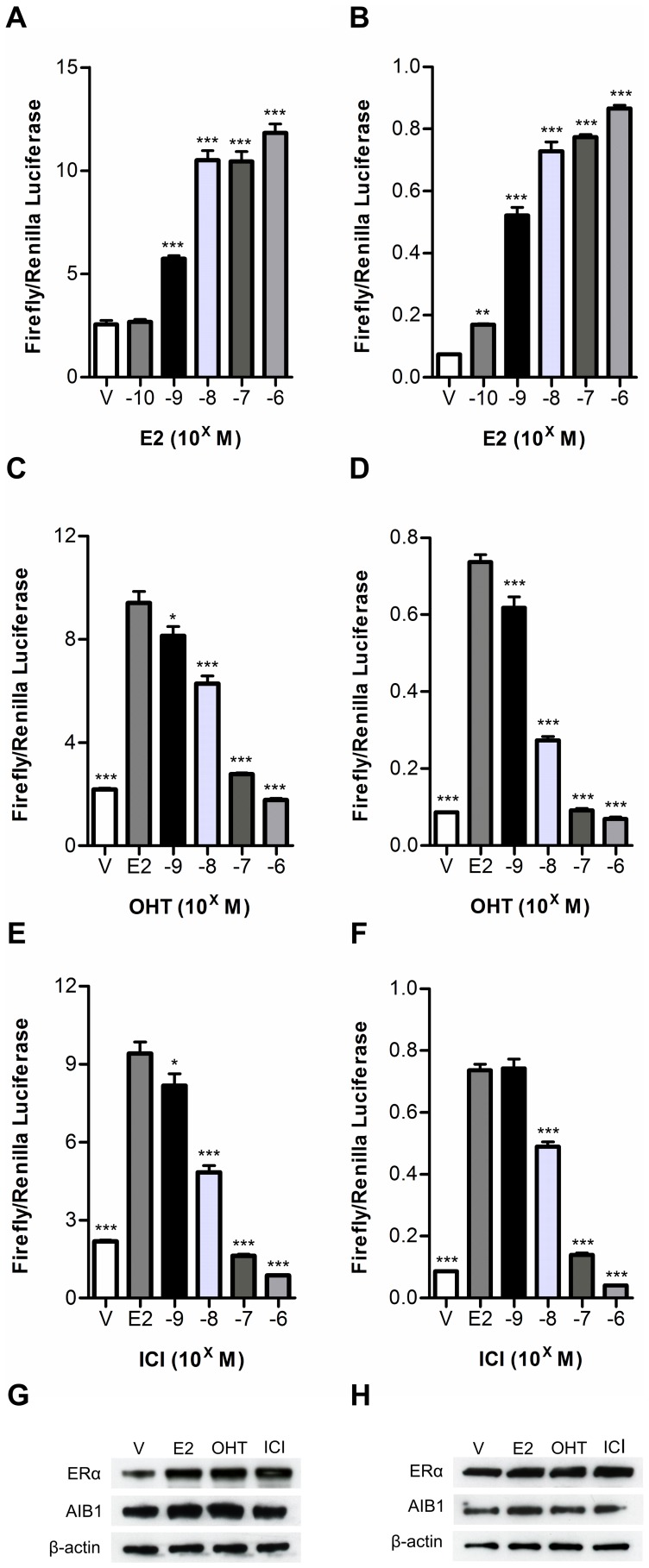
Estrogen and anti-estrogen regulation of ERα-AIB1 mediated luciferase fragment complementation. 293 cells were transiently transfected with N-S-AR and EL-S-C (A, C, E, and G) or N-EL and AR-S-C (B, D, F, and H). (A, B) Transfected cells were treated with vehicle (V) or increasing concentrations of E2 for 48 hours prior to quantification of luciferase activities. (C–F) Transfected cells were treated with vehicle (V) or 1 nM E2, in the absence (E2) or presence of increasing concentrations of OHT (C, D) or ICI (E, F) for 48 hours. In all cases data are expressed as firefly luciferase activity normalized to *Renilla* luciferase activity (± SEM of triplicates). ANOVA was used to determine statistical significance relative to vehicle (A,B) or E2 treatment (C–F; *** p≤0.001, ** p≤0.01, * p≤0.05). (G, H) Lysates from cells treated with vehicle or 1 μM E2, OHT or ICI for 48 hours were immunoblotted using antibodies for ERα, AIB1 or β-actin.

## Methods

Unless otherwise stated, all chemicals, enzymes and reagents were purchased from Sigma Aldrich.

### Plasmids and cloning of split luciferase constructs

The ER and AIB1 split luciferase constructs were generated by replacing the FRB and FKBP12 coding sequences in pcDNA-NLuc-FRB and pcDNA-FKBP12-CLuc (kindly provided by Professor S Gambhir, Stanford University [26]).

**Figure 4:**
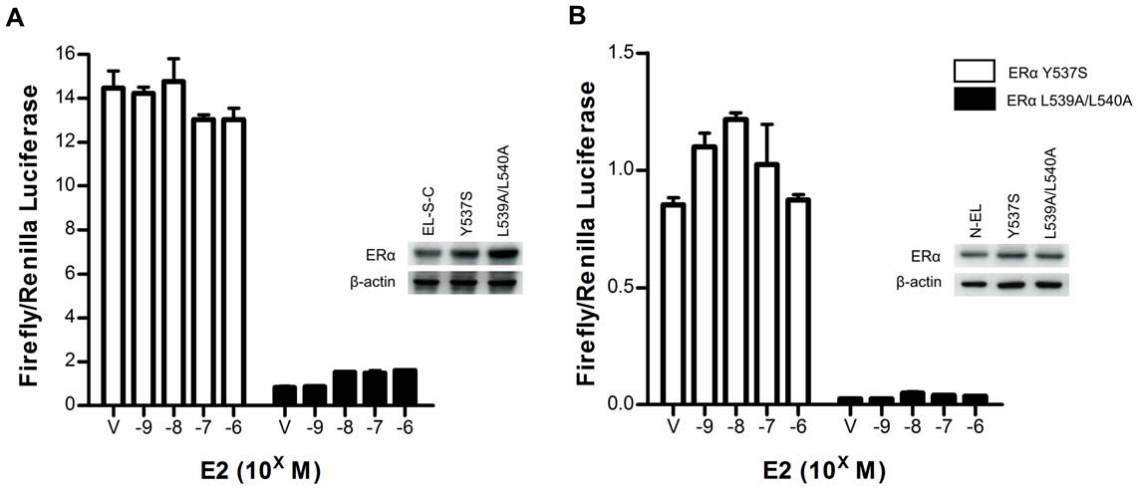
AIB1-ERα mediated luciferase fragment complementation is modulated by mutations in helix 12 of the ER-LBD. 293 cells were transiently transfected with N-S-AR (A) or AR-S-C (B) and reciprocal ER-LBD fusion constructs which were mutated to be constitutively active (Y537S; white bars) or incapable of interacting with AIB1 (L539A/L540A; black bars). Cells were treated with vehicle (V) or increasing concentrations of E2 for 48 hours prior to quantification of luciferase activities. Graphs indicate the ratio of firefly to *Renilla* luciferase activity (± SEM of triplicates). Western blots using antibodies for ERα were used to determine expression levels of the ER-LBD fusion protein mutants.

**Figure 5:**
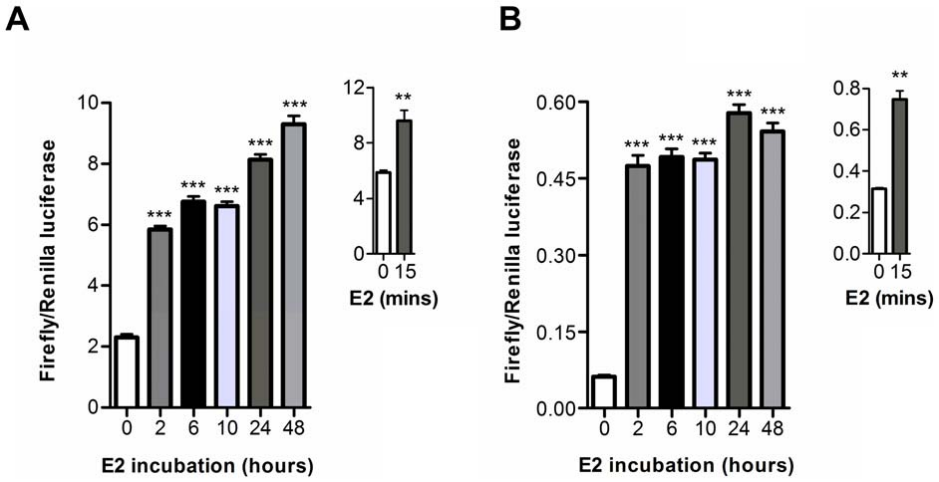
Time-course of E2 induced ERα-AIB1 luciferase fragment complementation. 293 cells transiently transfected with N-S-AR/EL-S-C (A) or N-EL/AR-S-C (B) were treated with vehicle or 1 μM E2 for 2–48 hours prior to quantification of luciferase activities. Inset graphs indicate change in luciferase activity after 15 minutes E2 incubation. Graphs show the ratio of firefly luciferase relative to *Renilla* luciferase activity (± SEM triplicates). ANOVA was used to determine statistical significance relative to vehicle (*** p≤0.001, ** p≤0.01).

*pcDNA-NLuc-ER-LBD (N-EL):* pcDNA-NLuc-FRB encodes amino acids 1–398 of the firefly luciferase in frame with the coding sequence of human FK506 binding protein FKBP12 rapamycin binding domain (FRB), with a *Bam*HI site separating the NLuc and FRB sequences and an *Xho*I site located 3′ to the FRB sequences. Sequences encoding amino acids 282–595 of human ERα comprising the LBD were PCR amplified using oligonucleotides having the sequence 5′-AGATCGGATCCTCTGCTGGAGACATGAGAGCTGCC-3′ and 5′-TGATCCTCGAGTCAGACTGTGGCAGGGAAACCCTCTGCC-3′ (*Bam*HI and *Xho*I sites underlined), using pSG5-ERα (HEG0; kindly provided by Prof P Chambon, Strasbourg, France [27]) as the template. The PCR fragment was purified using a Qiagen PCR product purification column, digested with *Bam*HI and *Xho*I and cloned into pcDNA-NLuc-FRB which had been digested with *Bam*HI/*Xho*I and dephosphorylated using a thermosensitive alkaline phosphatase (Thermo Scientific) to prevent recircularization. *pcDNA-NLuc-AIB-RID (N-AR):* N-AR was generated as described for N-EL above, by cloning of the AIB1-RID (amino acids 580–839) using pcDNA-AIB1 (kind gift of Prof P Meltzer, National Cancer Institute, Maryland [20]) as a template. For PCR, primers with sequences 5′-AGATCGGATCCGTGGAGAGTTCAATGTGTCAGTC-3′, 5′- CAGACCTCGAGCTACAAACCCAGAGAATTAGTTCCTT-3′ (*Bam*HI and *Xho*I sites underlined) were used. *pcDNA-ERα-LBD-CLuc (EL-C):* pcDNA-FKBP12-CLuc encodes human FK506 binding protein 1A (FKBP12) N-terminal to sequences encoding amino acids 394–550 of firefly luciferase, with a *Nhe*I site 5′ to the FKBP12 sequence and a *Bam*HI site between the FKBP12 and CLuc sequences. The pcDNA-CLuc fragment purified following *Nhe*I/*Bam*HI digestion of pcDNA-FKBP12-CLuc was ligated with the ERα LBD amino acids 282–595 generated by PCR amplification of pSG5-ERα using primers with the sequences 5′-AGATCGCTAGCATGTCTGCTGGAGACATGAGAGCTGCC-3′, 5′-TGATCGGATCCGACTGTGGCAGGGAAACCCTCTGCCTCCCCCGT-3′ (*Nhe*I and *Bam*HI sites are underlined) to generate the EL-C construct. *pcDNA-AIB1-RID-CLuc (AR-C):* AR-C was generated as was EL-C, using the product generated from PCR amplification of pcDNA-AIB1 using primers with the sequences 5′-AGATCGCTAGCATGGTGGAGAGTTCAATGTGTCAGTC-3′, 5′- TGATCGGATCCCAAACCCAGAGAATTAGTTCCTTG-3′ (*Nhe*I and *Bam*HI sites are underlined). All constructs were verified by sequencing.

**Figure 6:**
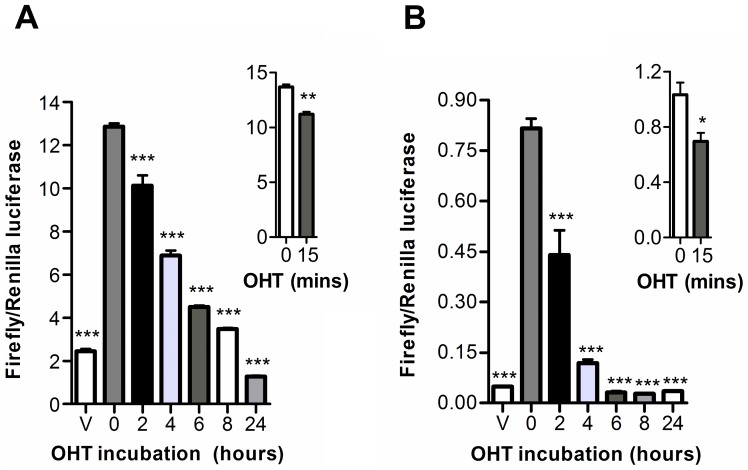
Time-course of OHT modulation of E2 induced ERα-AIB1 luciferase fragment complementation. 293 cells transiently transfected with N-S-AR/EL-S-C (A) or N-EL/AR-S-C (B) were treated with vehicle or 1 nM E2 for 24 hours prior to the addition of 1 μM OHT for 2–24 hours. Inset graphs indicate change in luciferase activity after 15 minutes OHT incubation. Graphs show the ratio of firefly luciferase relative to *Renilla* luciferase activity (± SEM triplicates). ANOVA was used to determine statistical significance relative to E2 treatment (C,D; *** p≤0.001, **p≤0.01, * p≤0.05).

*Spacer insertion:* Oligonucleotides having the sequences 5′- GATCCGGTGGAGGCGGTTCAGGCGGAGGTGGCAGCGGTACCC-3′ and 5′- GATCGGGTACCGCTGCCACCTCCGCCTGAACCGCCTCCACCG-3′, incorpo-rating a *Kpn*I site (underlined) and *Bam*HI overhangs, were hybridised in a 10 mM Tris, 50 mM NaCl, 1 nM EDTA, pH 8.0 reaction buffer to 80°C and cooled slowly. N-AR, N-EL, EL-C and AR-C constructs were digested with *Bam*HI and ligated with the hybridized oligonucleotides. Positives were identified by digestion with *Kpn*I, the number of copies inserted and their orientation was determined by sequencing. The constructs used in this article contain two tandem copies of the oligonucleotide spacer, giving the sequence GSGGGGSGGGGSGTRSGGGGSGGGGSGTRS between the fusion protein constituents (where G is glycine, S is serine, T is threonine and R is arginine).

**Figure 7:**
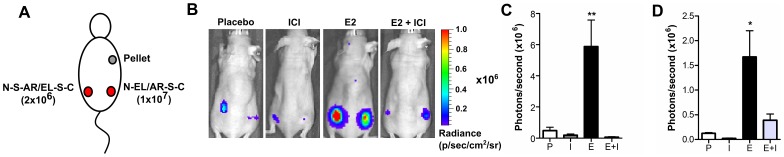
*In vivo* imaging of ERα-AIB1 mediated luciferase fragment complementation. 2×10^6^ N-S-AR/EL-S-C and 1×10^7^ N-EL/AR-S-C transfected 293 cells were subcutaneous implanted into the left and right flanks, respectively, of nude mice which had 0.72 mg/60 day E2 or placebo pellets implanted 7 days previously (A). Mice were treated with 5 mg ICI or vehicle (castor oil) at the time of cell implantation and 5 days before. 48 hours after cell implantation, mice were imaged with 150 mg/kg D-luciferin. Representative images (B) and quantitative data (± SEM, n = 4) are shown for N-S-AR/EL-S-C (C) and N-EL/AR-S-C (D) for each treatment group (Placebo (P) =  placebo and vehicle, ICI (I) =  placebo and ICI, E2 (E) =  E2 and vehicle, E2+ICI (E+I) = E2 and ICI). ANOVA indicates that the mean of the E2 treated groups was significantly different to all other groups (**p≤0.01, * p≤0.05).

*ERα Y537S and L539A/L540A mutants:* Mutations were introduced in EL-S-C and N-EL constructs using the QuickChange Site-Directed Mutagenesis kit (Agilent Technologies), according to the manufacturer’s methods. The primers used for mutagenesis had sequences 5′- TGGTGCCCCTCTATGACGCGGCGCTGGAGATGCTGGACG-3′, 5′ CGTCCAGCATCTCCAGCGCCGCGTCATAGAGGGGCACCA-3′ for the L539A/L540A mutant and 5′-AAGAACGTGGTGCCCCTCTCTGACCTGCTG-3′ and 5′-CAGCAGGTCAGAGAGGGGCACCACGTTCTT-3′ for the Y537S mutants. Mutations were verified by sequencing.

### Cell culture, transfection and luciferase assays

293H cells (Invitrogen) were routinely maintained in DMEM containing 10% FCS. For experiments in which E2 or anti-estrogens were added, the cells were cultured for 72 hours in phenol red-free DMEM supplemented with 5% dextran-coated charcoal stripped FCS and were maintained in this medium for the duration of the assay. 17β-estradiol (E2), 4-hydroxytamoxifen (OHT) and ICI 182,780 (ICI; Tocris Bioscience, Bristol, UK) were prepared in ethanol and an equal volume of ethanol was added to vehicle controls. 293 cells were transfected with 75 ng of each split luciferase plasmid, together with 50 ng of the *Renilla* luciferase reporter pRLTK (Promega) using Lipofectamine 2000 (Invitrogen). 6 hours post-transfection, the transfection mixture was removed and replaced with fresh medium containing E2, anti-estrogens or vehicle. Firefly and *Renilla* luciferase activity was quantified using the Dual Glo kit (Promega). Luminescence was captured using a Topcount Luminometer (Perkin Elmer).

### Lysate preparation and western blotting

Lysates were prepared using RIPA buffer with protease inhibitor cocktail and quantified by BCA assay (Thermo Scientific). Immunoblotting was performed using antibodies for ERα, AIB1 (sc8002 and sc9119, respectively; Santa Cruz Biotechnology) and β-actin (ab6276; Abcam).

### *In vivo* experimentation and ethics statement

Animal studies were performed in accordance with the UK Animal (Scientific Procedures) Act 1986 and National Cancer Research Institute guidelines [28] within a Designated Establishment under the 1986 Act (the Central Biomedical Services Unit at Imperial College London). The work was done under UK Home Office Project Licence 70/7113. Mice were maintained in individually ventilated cages with environmental enrichment. Procedures were performed with anaesthetic and post-operative analgesics. The studies were designed to detect changes in protein-protein interactions before palpable lesions developed (humane end point). Mice were sacrificed by a Schedule 1 approved method (dislocation of the neck) at the end of the experiment.

100 μl of PBS-cell suspension was subcutaneously injected into the flank of isofluorane anaesthetized 4–6 week old female nu/nu BALB/c mice (Harlan), 7 days following subcutaneous implantation of 0.72 mg/60 day release E2 or placebo pellets (Scientific Research of America). Mice were treated twice with 5 mg of ICI by subcutaneous administration of 100 μl of Faslodex (AstraZeneca) into the scruff at the time of, and 5 day prior to, cell implantation. An equal volume of castor oil was administered to control animals. ICI was used as the *in vivo* anti-estrogen because previous studies have indicated OHT metabolism in the mouse is considerably different to humans and rats, resulting in altered concentrations of tamoxifen and its major metabolites [29]–[32].

*In vivo* imaging was conducted using the IVIS 100 (Caliper Life Sciences). 48 hours after cell implantation, 150 mg/kg D-luciferin potassium salt in 100 μl sterile PBS was injected intraperitoneally and the animals were imaged after 15 minutes. Photon emission was quantified using the Living Image Software (Caliper Life Sciences); regions of interest (ROI) of a standard area were used to quantify the total photons/second (flux) emitted after subtraction of background ROI values.

### Statistical analysis

Statistical significance was determined by t-test or analysis of variance (ANOVA) using Prism 5.01 (Graphpad Software, California, USA). Tukey or Dunnett's multiple comparison post tests were used to compare treatment groups.

## Results

### Construct optimization

In order to determine if a split luciferase assay can be used to report on ERα-AIB1 interaction, luciferase fragment fusion protein constructs were generated in which the ER-LBD was fused to NLuc or CLuc, to give N-EL and EL-C, respectively (Figure 2A). Complementary constructs encoding the AIB-RID were generated for each of the ER-LBD constructs. Transient transfection of 293 cells showed that the addition of 17β-estradiol (E2) stimulated increased luciferase activity compared to vehicle, indicating that it is possible to achieve firefly luciferase reconstitution with both ER-LBD/AIB-RID construct sets (Figure 2A–C). There were, however, differences in the luciferase activity observed with N-EL/AR-C compared with N-AR/EL-C. The N-AR/EL-C construct pair demonstrated more than 10 fold higher luciferase reconstitution than the N-EL/AR-C pair, although the fold increase in luciferase activity upon addition of E2 was greater for N-EL/AR-C.

It has previously been suggested that inclusion of a flexible spacer between fusion protein constituents can reduce the deleterious effects on protein folding or function that might occur as a result of the unorthodox protein environment within a fusion protein [33], [34]. Accordingly, we investigated the effect of inserting a spacer sequence (-S-) between the ER-LBD or the AIB-RID and the luciferase fragment (Figure 2A–C). Inclusion of the flexible spacer did not universally improve complementation; the spacer increased or decreased luciferase reconstitution depending on the specific constructs. Where inclusion of the fusion protein did enhance complementation, it had little effect on the sensitivity of construct pairs (i.e. fold induction) because it tended to increase luciferase reconstitution equally in the presence and absence of E2.

From this initial assessment, we chose two construct pairs, N-S-AR/EL-S-C and N-EL/AR-S-C for further characterization. N-EL/AR-S-C exhibited the highest fold increase with E2, and might, therefore, be expected to provide the most sensitive means of assessing ERα-AIB1 interaction. However, because the overall luminescence intensity with this construct pair was relatively low, which could be problematic for imaging ERα-AIB1 interaction *in vivo,* where signal intensity reduces as function of tissue depth, we also chose to further characterize the N-S-AR/EL-S-C construct pair, which indicated the greatest overall luminescence.

### Estrogen and anti-estrogen regulation of ER-LBD/AIB-RID split luciferase interaction

Incubation of 293 cells transiently transfected with either N-S-AR/EL-S-C or N-EL/AR-S-C with increasing concentrations of E2 increased luciferase fragment complementation, up to the maximum concentration tested of 10^−6^M E2 (Figure 3A–B). With both construct pairs, approximately 50% of maximal activity was observed with 10^−9^M E2, which is consistent with the dissociation constant (Kd) of E2 bound ERα that is generally reported to be in the range of 10^−10^ to 10^−9^M E2 [35], [36]. The peak ERα-AIB1 interaction observed with 10^−6^M E2 using the ER-LBD/AIB-RID split-luciferase reporters, is also in agreement with GST-pull down assays investigating ERα-AIB1 interactions, which report maximal interaction in the presence of 10^−6^M E2 [37]. The SERM 4-hydroxytamoxifen (OHT) and complete ERα antagonist ICI 182, 780 (ICI) inhibited the E2 stimulated split luciferase complementation in a dose dependent manner (Figure 3C–F) and in a manner that is consistent with the relative binding affinities of ERα for these ligands [38], [39]. Western blotting indicated that the expression of all four of the fusion proteins was unaltered by treatment with the different ligands (Figure 3G–H).

### Mutant ER-LBD-luciferase fragment fusion proteins

To further demonstrate the specificity of the ER-LBD/AIB-RID split luciferase assay, mutant ER-LBD fusion proteins were produced. Two mutants were produced for each construct pair: one in which leucine residues 539 and 540 of helix 12, which are critical for coactivator recruitment, are replaced by alanine [40]; and one in which tyrosine 537 at the N-terminus of helix 12 to is mutated to serine to create a constitutively active ERα, which recruits coactivators in an E2-independent manner [41], [42]. Transfection of the constitutively active ER-LBD-Y537S constructs with their reciprocal AIB-RID construct produced high luciferase activity in an E2-independent manner, consistent with its described activity. Conversely, the ER-LBD-L539A/L540A mutants produced consistently low luciferase activity irrespective of E2 concentrations (Figure 4). Western blotting demonstrated that the poor luciferase complementation for this mutant was not due to lack of expression.

### Reporter dynamics

Having established that the ER-LBD/AIB-RID split luciferase assay is modulated by E2, OHT and ICI in a manner consistent with their described activities, we investigated the assay kinetics. Time-course studies indicated that luciferase fragment complementation increased with greater durations of E2 incubation (Figure 5A–B). Depending on the construct pair, 50–80% of maximum reporter activity was observed after 2 hours ligand incubation. Further experiments indicated that an E2 stimulated increase in luciferase activity could be observed after just 15 minutes of ligand incubation (inset graphs).

We proceeded to determine whether the split luciferase assay was capable of reporting on the dynamics of protein-protein interactions by investigating the impact that addition of the competitive inhibitor OHT had on the luciferase signal from E2 stimulated cells. Two hours after OHT addition, a 20–50% reduction in luciferase activity was observed (Figure 6 A–B). Again, further experiments indicated that changes in the luciferase signal of both construct pairs could be detected within 15 minutes of OHT addition (inset graphs).

### *In vivo* imaging of ERα-AIB1 interaction

To determine the sensitivity of the split luciferase constructs for *in vivo* studies, N-S-AR/EL-S-C and N-EL/AR-S-C transfected cells were subcutaneously implanted into the left and right flank, respectively, of mice treated with E2 or placebo pellets (Figure 7A). Imaging both reporters in each subject minimized the number of animals required for experimentation and facilitated a direct comparison of results. The number of cells implanted was adjusted in order to reduce differences in signal intensity between the reporters. Forty-eight hours after cell implantation, mice were imaged and an E2-induced increase in luciferase activity could be detected with both N-S-AR/EL-S-C and N-EL/AR-S-C transfected cells (Figure 7B–C). Furthermore, administration of the anti-estrogen ICI modulated the E2 stimulated luciferase activity observed. Interestingly, the degree of signal reduction differed between the two cell populations, which could indicate subtle differences between the reporters *in vivo*.

## Discussion

The estrogen signaling pathway is an attractive therapeutic target for the development of novel therapies for breast cancer and other estrogen related disorders. As our appreciation of the signaling pathway has developed, so has our appreciation of the genomic actions of ERα at gene promoters which do not contain direct ER binding sites (i.e., EREs). For example, E2 can stimulate the transcription of cMyc and IGF-I even though neither gene contains an ERE within its promoter. This indirect ERα stimulated gene expression, in common with direct ERE based gene expression, has been shown to be associated with the binding of coactivator proteins, including AIB1 [17]. As such, imaging such ERα-coactivator interactions, which are common requirement for ERα stimulated transcription, is an attractive means for determining ERα genomic activity. Previous efforts to image such interactions have been fluorescence based, and so have had limited capacity for non-invasive imaging in living subjects [43]–[46]. Here we have demonstrated that a split firefly luciferase assay can be used to non-invasively image the association of ERα with AIB1. The use of a bioluminescent, rather than fluorescent, reporter protein enables the method to be used for non-invasive imaging of ERα-AIB1 interactions in living subjects as well as *in vitro.* The ability to transfer the split luciferase assay from a relatively high throughput *in vitro* screening device to an *in vivo* validation tool is a significant advantage of the method.

By producing a range of different ERα-AIB1 split luciferase constructs, complimentary fusion proteins that exhibited more than 10-fold stimulation in luciferase activity with E2, were identified. Previous studies have highlighted the importance of producing a range of constructs for comparison. It has been shown that altering the specific protein fragments within fusion proteins, and altering their relative positions, can change the complementation observed between the same luciferase fragments [26]. This variability, which is thought to stem from the individual protein environment produced within a specific fusion protein, was apparent in the constructs that we produced; minor differences between N-S-AR/EL-S-C and N-EL/AR-S-C were apparent in many of the experiments conducted.

Methods which facilitate real-time imaging of ERα activity have considerable potential for the development of new drugs that target estrogen receptors. Studies with the ER-LBD/AIB-RID split luciferase molecular sensors indicated an excellent potential for imaging ERα-AIB1 protein-protein interaction kinetics. With both construct pairs, alterations in luciferase activity could be detected within 15 minutes of E2 addition, although longer incubations were required to reach a signal plateau. Previous studies investigating the kinetics of complementation between split luciferase fragments have indicated that maximal complementation is generally observed within 15 minutes of exposure to the interaction inducer [47]–[50]. This suggests that the incremental increase in signal observed in the ERα-AIB1 split luciferase assay is a reflection of ERα-AIB1 interaction kinetics, not a limitation of complementation between the luciferase fragments. Immunoprecipitation experiments have also indicated that the interaction between endogenous ERα and AIB1 proteins in MCF7 cells increases over a number of hours [51]. In addition to imaging ERα-AIB1 association, the split luciferase method described also indicated an excellent potential for imaging dissociation following anti-estrogen administration. With both construct pairs, modulation of the luciferase signal could be detected within 15 minutes of OHT addition, although full signal ablation was not observed for several hours. The timeframe of signal ablation observed with the ERα-AIB1 split luciferase assay is broadly consistent with a similar assay studying the dissociation of the androgen receptor from an LXXLL motif, which indicated a full signal ablation 2 hours after ligand withdrawal [52].

In the present study, all experiments have been performed by transient transfection of 293 cells, which express little or no endogenous ERα or AIB1. This has facilitated characterization of the assay with minimal interference. However, expression of the fusion proteins in E2 responsive cell lines may produce subtly different interaction patterns because of the active signaling pathways in different cellular contexts. Studies in transgenic mouse models indicate that the magnitude and timeframe of an E2 induced signal can differ depending on the specific tissue being studied [53], [54] and further studies will be required to determine whether the split luciferase constructs described here report on tissue specific ERα-AIB1 interactions. If this proves to be the case, then the method could be of great value in the screening of novel SERMs.

The ERα-AIB1 split luciferase method detailed in this report is a novel method for imaging activation of the estrogen signaling pathway. It is ERα specific and enables a broad appreciation of ERα genomic signaling to be achieved *in vivo*. It is anticipated that the method will be of great value in the identification of novel therapies aimed at the estrogen signaling pathway. The method is particularly applicable to the discovery of ERα specific ligands and to the identification of next generation compounds which aim to block ERα-coactivator interaction [55]–[58]. Furthermore, since differential ERα-coregulator interactions are understood to be central to the tissue specific actions of SERMs, it is hoped that the method presented could also form the basis of a novel method for the identification of new tissue specific ERα ligands.
